# Wisdom and value orientations: Just a projection of our own beliefs?

**DOI:** 10.1111/jopy.12530

**Published:** 2019-12-22

**Authors:** Judith Glück, Bianca Gussnig, Sarah M. Schrottenbacher

**Affiliations:** ^1^ Department of Psychology University of Klagenfurt Klagenfurt Austria

**Keywords:** people's conceptions of wisdom, Portrait Value Questionnaire, values, wisdom

## Abstract

**Objectives:**

This paper investigated which value orientations (1) people associate with wisdom and (2) are actually correlated with measures of wisdom. Conceptions of wisdom suggest benevolence and universalism as likely candidates.

**Method:**

In Study 1, 160 university students reported their political orientation and completed a value survey for themselves and a very wise person; Study 2 used the same approach with a more diverse sample (*N* = 187). In Study 3, 170 participants completed a value survey and six measures of wisdom. In Study 4, 356 participants completed a wisdom measure and filled out a value survey for themselves and a very wise person.

**Results:**

People consistently believed that wise individuals value benevolence, universalism, and self‐direction most; they also imagined wise individuals to be more universalistic but also more respectful of tradition than themselves. Several wisdom measures were uncorrelated with values; the positive correlations that were found were with benevolence, universalism, self‐direction, and respect for traditions.

**Conclusions:**

Most people believe that wise individuals are concerned about the well‐being of others, have respect for cultural, religious, and individual differences and traditions, and care deeply about self‐direction, fairness, and equality as fundamentals of human society. Whether these relationships are also found empirically depends on which measure of wisdom is used.

## 1 INTRODUCTION

The Austrian province of Carinthia, in which our university is located, was long considered a stronghold of right‐wing nationalist politics. In 2008, we happened to launch a media call for wisdom nominations not long after province governor Jörg Haider, a widely known right‐wing politician, had died in a self‐caused car crash in 2008. When we received nominations of people who had been close to him as exemplars of wisdom, we began to wonder about the relationship between wisdom and ideological values. We would never have considered Haider or his political sidekicks, who were as well‐known for corruption as for fomenting nationalism and xenophobia, as wise in any sense. But to what extent were we just projecting our own worldviews into wise individuals? Does a right‐wing nationalist consider Haider as just as wise as other people consider Gandhi or Mandela?

Wisdom is one of the great ideals that people strive to achieve. When we imagine a wise person, we imagine the person we hope to become, a person who has grown into a source of advice and care for others––and, presumably, a person who shares our fundamental beliefs and values. To what extent are wisdom researchers' ideas of wisdom‐related values influenced by their own ideologies? While most psychological conceptions of wisdom do not explicitly include value orientations, some of their components are clearly value‐laden. Drawing on Shalom Schwartz's theory of basic values (e.g., Schwartz, 1994, 2012), the self‐transcendent values of benevolence (caring for the welfare of one's group) and universalism (caring about the world at large, including nature) would seem particularly relevant to wisdom. Several wisdom conceptions explicitly include concern for the well‐being of others and a larger common good (e.g., Ardelt, 2003; Sternberg, 1998) or awareness of the relativity of one's own values and tolerance for other worldviews (e.g., Baltes & Staudinger, 2000; Grossmann et al., 2010) as components of wisdom. In an expert survey of wisdom researchers, concern for a common good and value relativism and tolerance were among the characteristics most closely associated with wisdom (Jeste et al., 2010). But are these ideas about wisdom shared by people who do not endorse these values as much as many psychologists do? For example, do people who do not consider universalism as an important value for themselves still share our conviction that wise individuals care about the world at large? Jonathan Haidt has pointedly argued that the long‐term emphasis of moral psychology on justice and care was to some degree a projection of researchers' liberal ideologies and that in many other parts of the world, as well as among conservatives in Western countries, other aspects of morality such as respect for authority, in‐group loyalty, and purity or sanctity, are considered as equally important (Haidt, 2008). Could wisdom researchers have fallen prey to a similar overgeneralization when they proposed wisdom components such as value relativism and tolerance (Baltes & Staudinger, 2000)? This paper aims to answer this question by taking two different approaches. First, we investigate to what extent people ascribe their own value orientations to wise individuals. Second, we investigate which actual value orientations are correlated with wisdom. In other words, this research looks at both the value orientations that people outside academia associate with wisdom and the value orientations that wise individuals actually have.

In Studies 1, 2, and 4, we examined the relationship between people's own value orientations and those that they ascribed to wise individuals. The main goal of this research was to decide between two hypotheses: if aspects like benevolence and universalism are generally viewed as characteristics of wisdom (at least in the Western world), then even people who do not endorse these values themselves should believe that wise individuals endorse them. If they are just projections of our own values, then those people should imagine wise individuals as being just as, or perhaps even more, nonbenevolent or nonuniversalistic than they are.

In Studies 3 and 4, we also analyzed actual relationships between value orientations and wisdom. Little earlier research has investigated this question. Kunzmann and Baltes (2003) found that wisdom was positively correlated to striving for self‐understanding and personal growth and to other‐oriented goals (well‐being of friends, societal engagement, and ecological protection) and negatively correlated to striving for a pleasurable life. Webster (2010) largely replicated these findings with a different measure of wisdom, except for a zero correlation with striving for a pleasurable life. Le (2008) found that values related to conservation (security, tradition, and conformity) were negatively related to wisdom in both a European‐American and a Vietnamese‐American sample. To get a more detailed picture and to examine consistency with people's views, Study 3 measured the full set of Schwartz's (1994, 2012) values and related them to six different measures of wisdom. In Study 4, combining the approaches of the earlier studies, participants from three age groups filled out a value survey for themselves and for a wise person as well as a measure of wisdom.

## 2 STUDY 1: VALUE ORIENTATIONS ASCRIBED TO WISDOM EXEMPLARS BY LEFT‐WING AND RIGHT‐WING PARTICIPANTS

Research on “laypeople's” theories of wisdom shows that most people describe wise individuals as having life experience and using it to provide advice and guidance for others, and as open to views diverging from their own and tolerant of individual differences (overview in Bluck & Glück, 2005). There are, however, individual differences in the centrality that people assign to some aspects of wisdom; for example, older adults view compassion as more central to wisdom than young adults do (Glück & Bluck, 2011). What people think about the values that wise individuals hold, and how these ideas relate to their own values, has not yet been investigated. Study 1 (Gussnig, 2016) investigated differences between the values that participants with right‐wing, neutral/center, and left‐wing political orientations ascribed to wise individuals.

Studies using Schwartz's circumplex model of human value orientations repeatedly found that individuals identifying with the political left (“liberals” in the North American terminology) had higher levels in the values of benevolence, universalism, and self‐determination and individuals identifying with the political right (“conservatives”) had higher levels in tradition, conformity, power, security, and achievement (Caprara, Schwartz, Capanna, Vecchione, & Barbaranelli, 2006; Caprara, Schwartz, Vecchione, & Barbaranelli, 2008; Piurko, Schwartz, & Davidov, 2011; Schwartz, Caprara, & Vecchione, 2010). Assuming that we would replicate these findings, the main research question of Study 1 was whether the two groups would also believe that wise individuals endorsed the respective values.

### 2.1 Method

#### 2.1.1 Participants

Study participants were recruited through listservs of Austrian universities and higher‐education colleges. Based on typical differences between different fields of study in the political orientations of their students, as reflected in student‐council elections, we tried to reach as many different universities and fields of study as possible. Eventually, a total of 160 students (68.1% women, age range 18 to 70 years, *M* = 26.9, *SD* = 9.2) from various fields of study completed the online survey, of whom 85 (53.2%) self‐identified as left‐wing (23) or left‐leaning (62), 52 (32.5%) as neutral/center, and 23 as right‐leaning (20), right‐wing (2), or extreme right‐wing (1). Thus, in spite of our efforts to reach right‐wing individuals, the distribution was highly unequal. However, we considered the sample of 23 right‐wing participants as sufficient for the first test of our hypotheses. In Study 4, the same analysis was conducted with a somewhat larger and more heterogeneous sample; results are reported there.

#### 2.1.2 Measures

The invitation email included a link to an online survey in which participants were first asked to report demographic data, including their political orientation on a 7‐point scale from “extreme left” to “extreme right.” They were also asked whether they had ever been in touch with scientific research on wisdom; those who agreed to this question were later excluded from the analyses. Afterward, they were presented with the most recent German version of the Portrait Values Questionnaire (PVQ‐RR; German version by Beierlein, Davidov, Schmidt, Schwartz, & Rammstedt, 2012). The PVQ‐RR includes three items for each of 19 human foundational values proposed by Schwartz et al. (2012, p. 669):

- Benevolence/caring (devotion to the welfare of in‐group members),
- Benevolence/dependability (being a reliable and trustworthy member of the in‐group),
- universalism/tolerance (acceptance and understanding of those who are different from oneself),
- universalism/concern (commitment to equality, justice, and protection for all people),
- universalism/nature (preservation of the natural environment),
- humility (recognizing one's insignificance in the larger scheme of things),
- conformity/rules (compliance with rules, laws, and formal obligations),
- conformity/interpersonal (avoidance of upsetting or harming other people),
- tradition (maintaining and preserving cultural, family, or religious traditions),
- security/personal (safety in one's immediate environment),
- security/societal (safety and stability in the wider society),
- face (security and power through maintaining one's public image and avoiding humiliation),
- power/dominance (power through exercising control over people),
- power/resources (power through control of material and social resources),
- achievement (success according to social standards),
- hedonism (pleasure and sensuous gratification),
- stimulation (excitement, novelty, and change),
- self‐direction/thought (freedom to cultivate one's own ideas and abilities),
- and self‐direction/action (freedom to determine one's own actions).

Each item is a one‐sentence description of an individual of the same gender as the participant who strongly endorses the respective value (e.g., “Protecting society's weak and vulnerable members is important to him” [universalism/concern]; “She wants people to admire her achievements” [achievement]). Participants rated each description on a 6‐point scale from “not like me at all” to “very much like me.” They first filled out the PVQ‐RR with respect to themselves. Then, they were asked to name the person whom they considered as most similar to an ideally wise person and to report at least three characteristics of this person. Afterward, they filled out the PVQ‐RR as they thought the wise person would fill it out.

Following the scoring guidelines for the PVQ‐RR (Beierlein et al., 2012), individual response tendencies were controlled by subtracting participants' individual averages across all items from their averages for each dimension, resulting in an average of zero over all dimension scores for each participant. This correction was performed separately for participants' reports of their own and the wise individual's values. Reliabilities were comparable to or higher than those reported by Schwartz et al. (2012), with Cronbach's alphas ranging from .58 for stimulation to .89 for tradition.

### 2.2 Results

#### 2.2.1 Participants' own values

**Figure 1:**
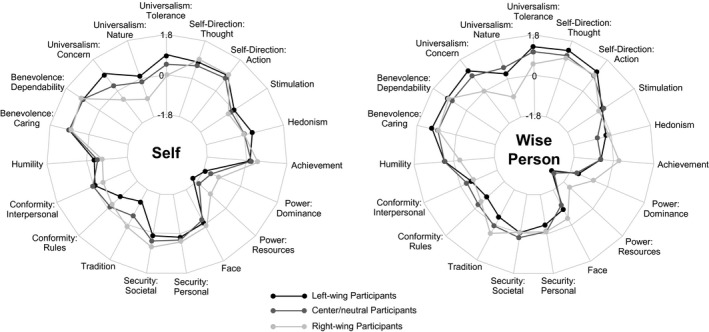
Study 1: Value orientations reported by left‐wing, center‐neutral, and right‐wing participants for themselves and the wisest person they know

Figure 1 shows the means of the three groups (left‐wing, neutral/center, and right‐wing) as they characterized themselves and their respective wisdom nominees concerning the 19 value dimensions, arranged according to the circular continuum of human values (Schwartz et al., 2012). As the figure shows, the general profiles of the three groups were relatively similar, with the highest means for the two self‐direction dimensions and benevolence and the lowest for the power dimensions.

Group differences were tested using univariate analyses of variance with post hoc Scheffé tests using the Bonferroni‐Holm correction for multiple group comparisons. Means, standard deviations, and effect sizes are displayed in Table 1, which also shows Spearman correlations between the value dimensions and political orientation. The significant differences in participants' own values replicated earlier research (Caprara et al., 2006, 2008; Piurko et al., 2011; Schwartz et al., 2010). For left‐wing and center/neutral participants, universalism/tolerance and universalism/concern were just as important as benevolence and self‐direction, followed by humility. Right‐wing participants also rated benevolence and self‐direction highest, followed by universalism/tolerance, achievement, and tradition. Universalism/nature was rated lower than the other two universalism dimensions by all three groups.

**Table 1:** Study 1: Means and standard deviations of left‐wing, center/neutral, and right‐wing participants in the 19 value dimensions (self and wise person) and correlations between value dimensions and political orientation

Value dimension	Left‐wing (*N* = 85)			Center/neutral (*N* = 52)			Right‐wing (*N* = 23)			Group difference+ (partial *η* ^2^)	Group difference+ (partial *η* ^2^)	*ρ*	*ρ*
	Self	Wise person	Diff. (Cohen's *d_z_*)	Self	Wise person	Diff. (Cohen's *d_z_*)	Self	Wise Person	Diff. (Cohen's *d_z_*)	Self	Wise Person	Self	Wise person
Stimulation	−.021 (.838)	.057 (.933)	−.070	−.164 (.735)	.136 (.972)	−.337	−.277 (.878)	−.072 (.973)	−.231	.014	.005	−.132	−.075
Hedonism	.348 (.863)	−.218 (1.188)	.512***	−.023 (.771)	−.621 (1.258)	.565***	−.016 (.996)	−.116 (.992)	.090	.045	.029	−.169	−.078
Achievement	.179 (.736)	−.539 (1.126)	.633***	.227 (.681)	−.544 (.913)	.792***	.491 (.655)	.275 (.891)	.253	.022	.073**	.138	.233**
											C = L < R		
Power/dominance	−1.691 (.980)	−1.398 (1.187)	−.227	−1.439 (.976)	−1.473 (1.339)	.027	−1.059 (1.158)	−.652 (1.339)	−.396	.046	.046	.221	.168
Power/resources	−2.009 (.959)	−2.469 (1.020)	.482***	−1.651 (1.135)	−2.352 (1.186)	.710***	−.958 (1.445)	−1.391 (1.308)	.297	.098***	.098***	.248**	.165
										L = C < R	L = C < R		
Face	−.076 (.861)	−.759 (1.029)	.564***	−.247 (.740)	−.954 (.851)	.760***	.056 (.610)	−.348 (.985)	.413	.017	.038	−.025	.082
Security/personal	.211 (.594)	−.367 (.799)	.655***	.317 (.554)	−.108 (.803)	.501**	.346 (.639)	−.058 (1.031)	.565	.010	.027	.128	.223
Security/societal	.140 (.785)	−.006 (.965)	.209	.362 (.610)	.219 (.775)	.180	.607 (.823)	.232 (.802)	.449	.050	.017	.269**	.175
Tradition	−1.154 (1.010)	−.371 (1.206)	−.686***	−.439 (1.104)	.072 (1.082)	−.465**	.114 (.962)	.449 (.809)	−.447	.177***	.070**	.444***	.307***
										L < C = R	L = C, C = R		
Conformity/Rules	−.785 (1.061)	−.771 (1.196)	−.013	−.176 (.929)	−.287 (1.187)	.097	−.262 (1.163)	−.464 (.998)	.150	.075**	.035	.270*	.176
										L = R, R = C			
Conformity/interpersonal	−.040 (.856)	−.563 (1.028)	.434***	−.003 (.838)	−.358 (.970)	.300	−.465 (.845)	−.696 (1.017)	.237	.033	.014	−.130	.048
Humility	−.334 (.858)	.382 (.962)	−.658***	−.503 (.645)	.366 (1.028)	−.928***	−.683 (1.261)	−.275 (1.179)	−.263	.021	.049	−.121	−.173
Universalism/nature	.085 (.972)	.214 (1.225)	−.109	−.164 (.945)	.514 (.886)	−.681***	−.958 (.970)	−.899 (.870)	−.052	.119***	.151***	−.341***	−.191
										R < C = L	R < L = C		
Universalism/concern	.944 (.565)	1.143 (.688)	−.309**	.259 (.686)	.873 (.832)	−.758***	−.494 (.969)	.000 (1.090)	−.484	.373***	.189***	−.639***	−.380***
										R < C < L	R < C = L		
Universalism/tolerance	.826 (.630)	1.214 (.678)	−.506***	.458 (.577)	1.001 (.698)	−.742***	−.045 (.944)	.449 (1.213)	−.574	.176***	.100***	−.398***	−.321***
										R < C < L	R < C = L		
Benevolence/care	.850 (.575)	1.104 (.616)	−.380**	.888 (.520)	.789 (.677)	.127	.839 (.633)	.797 (.715)	.062	.001	.055	.035	−.249**
Benevolence/dependability	.901 (.631)	.970 (.692)	−.106	.965 (.506)	.770 (.714)	.297	.926 (.607)	.957 (.708)	−.047	.002	.017	.027	−.076
Self‐direction/action	.870 (.706)	1.065 (.742)	−.230	.708 (.584)	.886 (.798)	−.205	.912 (.428)	.870 (.618)	.075	.016	.015	−.031	−.152
Self‐direction/thought	.756 (.705)	1.312 (.690)	−.644***	.625 (.569)	1.072 (.743)	−.544***	.926 (.657)	.942 (.697)	−.028	.022	.041	−.085	−.245**

*** *p* < .001;

** *p* < .01;

* *p* < .05.

Significance levels are Bonferroni‐Holm corrected. *ρ* = Spearman's Rho with political orientation.

^+^ For significant group differences, Scheffé test results are reported. For example, R = C < L: the right‐wing group and center/neutral group do not differ in means, but have a significantly lower mean than the left‐wing group.

Left‐wing participants had higher means than right‐wing participants in all three universalism dimensions, with the largest group difference in universalism/concern. Right‐wing participants had higher means than left‐wing participants in tradition and both power dimensions (although power still had the lowest mean of all values). Center/neutral participants had higher means than left‐wing participants in conformity/rules. In all other dimensions, they were intermediate between the left‐ and right‐wing participants, siding with the left‐wing participants on universalism/nature and power/resources and with the right‐wing participants on tradition.

#### 2.2.2 Values ascribed to wisdom nominees

The main question of the current research concerned the values that the participants ascribed to wise individuals. As Figure 1 and Table 1 show, the three groups agreed about the high importance of self‐direction and benevolence for their wisdom nominees (as well as for themselves). Left‐wing and center/neutral participants had higher means than right‐wing participants in the universalism dimensions, and right‐wing participants had higher means in tradition, power, and achievement. An interesting picture emerged when we compared participants' own means to those they ascribed to their respective wisdom nominees. Left‐wing participants described their wisdom nominees as even higher than themselves in universalism/tolerance and self‐direction/thought, but also as higher than themselves in humility and tradition. They also described their wisdom nominees as lower than themselves in security/personal, the self‐enhancement values of face, power/resources, and achievement, and in hedonism. Right‐wing participants had no significant differences between their own values and those they ascribed to a wise person. This was to some extent due to their small group size; descriptively, they had effect sizes similar to the two other groups on tradition, universalism/concern, and universalism/tolerance, where they imagined the wise person to be higher than themselves, and for the security dimensions, where they imagined them to be lower than themselves. Interestingly, while both the left‐wing and center‐neutral participants imagined the wise person to be higher than themselves in self‐direction/thought, there was no such difference for the right‐wing participants. Center/neutral participants largely paralleled the left‐wing participants in the differences between themselves and their wisdom nominees. The correlations between political orientation and the value dimensions were largely consistent with the group differences. The further to the right participants placed themselves on the political continuum, the higher were their scores in tradition and conformity/rules and the lower in universalism. Concerning the wisest person they knew, participants who placed themselves further on the right imagined this person to value tradition more and universalism, benevolence/care, and self‐direction/thoughtless.

### 2.3 Discussion

This study investigated differences between university students self‐identifying as politically left‐wing, neutral/center, and right‐wing in the values they endorsed themselves and those that they ascribed to the wisest person they knew. Concerning participants' own values, the differences between the three groups replicated earlier research (Caprara et al., 2006, 2008; Piurko et al., 2011; Schwartz et al., 2010): all three groups considered benevolence and self‐direction as most important and power as least important. However, left‐wing participants considered universalism (tolerance and concern) as equally important as benevolence and self‐direction, and they rated power and tradition as least important. For right‐wing participants, benevolence and self‐direction were followed by security, achievement, and tradition, and universalism/nature and universalism/concern were second‐lowest in importance after power. Center/neutral participants were largely intermediate between the two other groups. Thus, participants' own value orientations were consistent with their political attitudes.

Interestingly, however, these differences did not fully translate into participants' views of the values of wisdom nominees. In spite of differences in overall levels, all three groups believed that their respective wisdom nominees were more universalistic concerning tolerance and concern than themselves, but also more traditional than themselves and less concerned about their personal security. This finding suggests that there is an ideology‐independent component in the values that participants associate with wisdom: people imagine wise individuals to be more concerned about the needs of humanity at large, more tolerant of differences between individuals, and more respectful of cultural and religious traditions than they themselves are. Notably, universalism and tradition were the values where the right‐wing and left‐wing participants' own values were most divergent, suggesting that they assume wise individuals to take a more balanced stance than they do. One interesting finding was that universalism with respect to nature––that is, environmental protection––had a far lower mean than the two other components of universalism both concerning participants' own values and those they ascribed to wise persons. The data for this study were collected in 2016; at the time we are writing this (October 2019), this picture might look quite different due to the recent increases in people's awareness of the dangers of climate change.

Moreover, the general value profiles that the left‐wing and right‐wing participants drew of their wisdom nominees differed significantly in several aspects. Left‐wing and center/neutral participants believed that their wisdom nominees cared little about their own standing: they described them as high in humility and low in achievement, power, face, personal security, and conformity. Right‐wing participants also viewed their wisdom nominees as caring little about conformity, power, and face, but as relatively high in achievement and low in humility. These findings are reminiscent of McAdams et al.'s (2008) qualitative study of the idealized father figures of conservative and liberal Americans: while liberals described ideal fathers as empathetic, open, democratic, tolerant, and nurturant, conservatives descrived a more authoritative, discipline‐oriented ideal.

In sum, neither of our hypotheses was completely disconfirmed. While the value profiles that left‐wing and right‐wing participants reported for their wisdom nominees did differ in the same dimensions where their own values differed, neither group seemed to simply project their own values onto the wisdom nominees. Left‐wing participants described them as more tradition‐oriented and right‐wing participants described them as more universalistic than themselves. These findings suggest a general idea of wise individuals as combining concern for the world at large and acceptance of individual and cultural differences with respect for tradition––in other words, a wise individual may respect the traditions of his or her own cultural upbringing just as much as those of other people in other parts of the world. An interesting aspect that emerged as highly central both to participants' own values and those they ascribed to wise individuals was self‐direction. Having control of one's own life and freedom to decide was viewed as important both for oneself and for one's nominated wise person by participants of all political orientations.

Concerning the limitations of this research, one important question concerns the way we assessed which values participants ascribed to wise individuals. One could argue that asking them to fill out value scales as they thought a wise person would focuses too much on cognitive representations of wisdom and therefore does not reflect the more emotional way in which people experience wisdom in real life. We believe, however, that this problem mostly concerns value orientations that are not associated with wisdom. Participants may have had a hard time, for example, deciding how much a wise person would value hedonism or conformity. It seems unlikely to us that participants would have to think carefully about whether they believe that a wise person values benevolence or self‐direction. Still, future research might look into more naturalistic accounts of people's experiences with wise persons' value orientations, such as people's stories about their experiences with wisdom nominees (for an example of such an approach, see Montgomery, Barber, & McKee, 2002).

The generalizability of our findings is limited in at least three ways. First, participants were mostly university students. Second, they were thinking of a specific wise individual, which may have led them to draw less upon their general ideas about wisdom than upon their knowledge about that particular person. And third, participants were grouped according to political orientation, which may not always be based on clear, explicit individual value orientations; young people may have adopted their political orientations from family members or peers without much consideration. Study 2 aimed at resolving these shortcomings.

## 3 STUDY 2: VALUE ORIENTATIONS ASCRIBED TO IDEALLY WISE PERSONS

For Study 2 (Schrottenbacher, 2017), participants were broadly recruited through various online channels. Instead of thinking about a concrete individual they considered as wise, they were asked to imagine an ideally wise person and fill out the PVQ as this person would. Finally, the sample was not grouped according to political ideology, but differences were analyzed separately for each value dimension. Thus, the main aim of Study 2 was to investigate to what extent the similarities and differences identified in Study 1 would replicate if participants high and low in each value dimension were compared: for example, would even participants explicitly low in benevolence agree that wise individuals are benevolent?

### 3.1 Method

#### 3.1.1 Participants

Participants were recruited through various online channels, including snowball distribution on Facebook and a link to the study on the homepage of the magazine “Psychologie heute” (the German version of “Psychology Today”). The final sample consisted of 187 individuals (78.1% women) ranging in age from 15 to 75 years (*M* = 36.22 years, *SD* = 15.00). Education was relatively heterogeneous, with 31 participants (16.8%) having completed only the nine years of schooling compulsory in Austria, 62 (33.5%) having completed the equivalent of high school, and 87 (46.5%) having a college or university degree.

#### 3.1.2 Measures

Study 2 used the original 40‐item version of the Portrait Values Questionnaire (German version by Schmidt, Bamberg, Davidov, Herrmann, & Schwartz, 2007). The PVQ measures 10 value dimensions––self‐direction, power, universalism, achievement, security, stimulation, conformity, tradition, hedonism, and benevolence––using three to six items per scale. As in the PVQ‐RR, each item is a short description of an individual of the same gender as the participant who strongly endorses the respective value. Participants rate each description on a 6‐point scale from “not like me at all” to “very much like me.” Reliabilities in the current study ranged from .58 (tradition) to .87 (achievement). After reporting demographic information, participants first filled out the PVQ with respect to their own value orientations. Then, they were asked to imagine an ideally wise person and to take some time to think about what this person looks like, how he or she typically behaves, and what characterizes him or her. Next, they were asked to report the gender of the ideally wise person. Based on their response, they were presented with the male or female version of the PVQ and asked to fill it out as they thought the ideally wise person would. As in Study 1, individual response tendencies were controlled by subtracting participants' individual means across all items from the mean scores for each dimension.

### 3.2 Results

**Figure 2:**
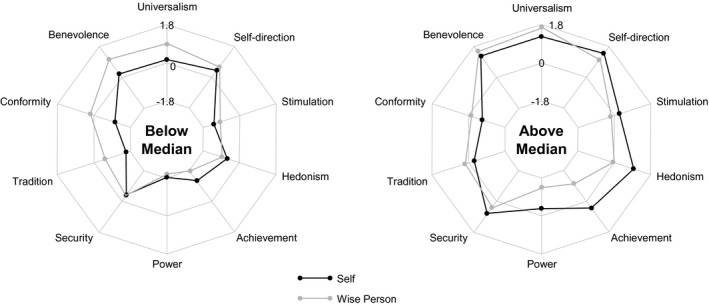
Study 2: Value orientations reported by participants above and below the median for each value dimension for themselves and an ideally wise person

For each of the basic values, the sample was split at the median of participants' personal endorsement of the value. Figure 2 and Table 2 display the means of the two groups for their personal values and their beliefs concerning an ideally wise person's values. As Figure 2 shows, the two light‐grey lines describing the ideally wise persons  suggest a highly consistent pattern across the two groups in spite of their differences in level (which were significant for all value dimensions not just––naturally, due to the median splits––concerning the participants themselves, but also concerning the ideally wise individuals). As Table 2 shows, the values that the participants ascribed to the wise individual differed significantly from their own values for most value dimensions. Participants described the ideally wise individuals as higher than themselves in benevolence, universalism, and tradition, and lower than themselves in achievement; participants high in power and hedonism also described wise individuals as lower than themselves in these two dimensions. The largest negative differences, with Cohen's *d*_s_ near or above 1, were found for benevolence, universalism, and tradition in the below‐median group, showing that participants low in these characteristics imagined an ideally wise person to be much higher in them than themselves. The largest positive differences were found for power and achievement in the above‐median group, showing that participants high in these characteristics imagined a wise person to be much lower in them than themselves.

**Table 2:** Study 2: Means and standard deviations of participants above and below the median in each dimension (self and wisdom nominee)

Value dimension	Low			High			Group difference (Cohen's *d_s_*)	Group difference (Cohen's *d_s_*)
	Self	Wisdom nominee	Difference (Cohen's *d_z_*)	Self	Wisdom nominee	Difference (Cohen's *d_z_*)	Self	Wisdom nominee
1 Stimulation	−1.286 (.559)	−.982 (.885)	−.301**	.244 (.563)	−.213 (1.058)	.446***	−2.727***	−.787***
10 Self‐direction	.386 (.376)	.609 (.617)	−.341**	1.374 (.368)	1.000 (.744)	.440***	−2.655***	−.579***
2 Hedonism	−.603 (.657)	−.866 (1.066)	.228	.951 (493)	−.062 (1.127)	.810***	−2.673***	−.733***
3 Achievement	−1.161 (.655)	−1.721 (.821)	.625***	.412 (.521)	−1.024 (1.169)	1.195***	−2.656***	−.691***
4 Power	−1.832 (.568)	−1.987 (.853)	.180	−.331 (.613)	−1.317 (.961)	1.032***	−2.539***	−.737***
5 Security	−.380 (.492)	−.320 (.715)	−.092	.747 (.391)	.347 (.547)	.727***	−2.533***	−1.047***
6 Tradition	−1.591 (.497)	−.541 (.201)	−.997***	−.276 (.590)	.201 (.838)	−.612***	−2.409***	−.779***
7 Conformity	−1.037 (.523)	.168 (.468)	−.426***	−.683 (.857)	−.104 (.738)	.329**	−2.427***	−.724***
8 Universalism	.132 (.559)	.871 (.835)	−.916***	1.223 (.314)	.1.633 (.508)	−.847***	−2.407***	−1.103***
9 Benevolence	.173 (.407)	1.035 (.618)	−1.243***	1.219 (.365)	1.481 (.600)	−.444***	−2.703***	−.733***

*** *p* < .001;

** *p* < .01. Significance levels are Bonferroni‐Holm corrected.

### 3.3 Discussion

The findings of Study 2 were consistent with those of Study 1 but clearer, as participants were grouped by their actual values rather than by political orientations. While participants' own value orientations certainly influenced the values they ascribed to wise individuals, these differences affected the level much more than the pattern of values that they associated with wisdom. Independent of their own value orientations, participants described an ideally wise individual as high in benevolence, universalism, and self‐direction and low in power and achievement. They also imagined an ideally wise individual to be higher than themselves, though not particularly high, in tradition, and lower than themselves in hedonism. For the other values––conformity, security, and stimulation––, the values ascribed to wise individuals were near the scale midpoint and less extreme than those of the participants. In both panels of Figure 2, the wise person's value pattern was shifted to the top in comparison to participants' own value patterns, showing again that people imagine wise persons to be higher in self‐transcendent values and lower in self‐protective values than themselves.

In sum, the picture of wise individuals that emerges from Studies 1 and 2 is quite consistent and mostly relates wisdom to Schwartz's dimensions of self‐transcendence and self‐direction: people generally seem to believe that wise individuals care both about members of their own group and about humanity at large and that they value the freedom to live a self‐directed life. At the same time, wise individuals are assumed to value cultural, religions, and familial traditions more than other people do. They are not assumed to care much about self‐enhancement, as manifested in power over others or personal achievement, or about their own security or pleasure.

This picture is quite consistent with earlier research investigating actual relationships between measures of wisdom and value orientations. Kunzmann and Baltes (2003) found that performance in the Berlin Wisdom Paradigm was positively related to an orientation toward insight and personal growth as well as to other‐enhancing orientations concerning the well‐being of friends, societal engagement, and ecological protection and negatively related to striving for a pleasurable life. Webster (2010), using the Self‐Assessed Wisdom Scale, largely replicated those findings except for a zero correlation with living a pleasurable life. Moreover, Le (2008) found negative relationships between wisdom and the meta‐value of conservation, which includes tradition. The negative relationship may, however, have been caused by other aspects of conservation such as security and conformity––a hypothesis to which we come back in Study 4. In Study 3, we moved our focus from investigating people's ideas about wise individuals to actually investigating how measured wisdom relates to Schwartz's value dimensions.

## 4 STUDY 3: ACTUAL RELATIONSHIPS BETWEEN WISDOM AND VALUE ORIENTATIONS

Studies 1 and 2 showed that people hold quite consistent beliefs about relationships between wisdom and value orientations. Study 3 tested whether these relationships are reflected in actual correlations between measures of wisdom and value orientations. If that is the case, wisdom should be positively related to universalism, benevolence, self‐direction, and tradition, and negatively to power and possibly to other self‐related values such as achievement, security, or hedonism. This study was the first to relate wisdom to all subdimensions of Schwartz's value scales, as most earlier research (Kunzmann & Baltes, 2003; Le, 2008; Webster, 2010) used less comprehensive value‐orientation measures. As method variance plays an important role in wisdom research (Glück et al., 2013), six different measures of wisdom––three self‐report measures and three open‐ended measures––were used. We assumed that correlations of wisdom measures with value orientations might reflect, to some extent, the value aspects that were part of the conceptions of the respective wisdom measures. For example, Ardelt's (2003) conception of wisdom, which underlies the Three‐Dimensional Wisdom Scale, puts some emphasis on compassion and concern for others, whereas the Berlin wisdom paradigm (Baltes & Staudinger, 2000) includes value relativism as one criterion for a wise response.

### 4.1 Method

#### 4.1.1 Participants

This study was part of a larger research project investigating the development of wisdom (see, e.g., Glück, Bluck, & Weststrate, 2018; Glück et al., 2013; König & Glück, 2014); data collection took place from 2009 to 2011. As wisdom is a rare phenomenon, we aimed to recruit a higher proportion of wise individuals by including wisdom nominees. As mentioned earlier, in the fall of 2008 calls were issued in local newspapers and radio programs in the Austrian province of Carinthia, asking people who knew a particularly wise person to contact the project team. Excluding self‐nominations, 82 individuals were nominated as wise, of whom 47 agreed to participate. Other participants were recruited through letters sent to about 1,600 Carinthians from a commercially available address list; a few older participants were contacted through students and colleagues. In total, 47 wisdom nominees and 123 other participants took part in the study; thus, the whole sample consisted of 170 people (90 women, 80 men). Age ranged from 19 to 95 years (*M* = 56.0, *SD* = 16.1); 64.7% of the participants were married or living with a partner, 23.8% had a university degree, and 38.8% were retired. Most participants came to the lab for two interview sessions and filled out some materials at home before and between the interview sessions; a few older participants were interviewed in their homes. Participants received € 70 (about $ 80) for their participation.

Because of resource limitations, the open‐ended wisdom measures were transcribed and rated only for the 47 wisdom nominees and 47 age‐ and gender‐parallel control participants. In this subsample, age ranged from 26 to 92 years (*M* = 60.3, *SD* = 15.8); 58.6% of the participants were married or living with a partner, 23.0% had a university degree, and 49.4% were retired.

### 4.2 Measures

#### 4.2.1 Schwartz Value Survey

After the first interview session, participants were given a German version of the Schwartz Value Survey (SVS; Schwartz, 1992; German version by Glöckner‐Rist, 2006) and asked to fill it out at home and bring it to the second session. The German SVS is a list of 57 values each described by a name and a brief explanation, such as “social justice (eliminating injustice, taking care of those in need)” for universalism, “respect for tradition (preservation of time‐honored customs)” for tradition, or “authority (the right to lead and make decisions)” for power. Participants rate to what extent they consider each value as a guiding principle in their lives. The 9‐point response scale is labeled at −1 (opposed to my principles), 0 (not important), 3 (important), 6 (very important), and 7 (extremely important). The 57 items refer to the same 10 value dimensions as the PVQ described in Study 2; Cronbach's alphas ranged from .64 (stimulation and tradition) to .81 (universalism; see Table 3). As recommended for the SVS, scores were corrected for individual response tendencies by centering each participants' item responses on his or her overall mean (Kusurkar & Croiset, 2015).

**Table 3:** Study 3: Correlations between wisdom measures and value dimensions

	Average wisdom rating/diff. event (*N* = 79–83)	Average wisdom rating/conflict (*N* = 79–83)	Berlin wisdom paradigm (*N* = 84–88)	3D‐WS (*N* = 151–158)	SAWS (*N* = 151–158)	ASTI (*N* = 150–157)
Stimulation (*α* = .64)	.015	.011	.153	.178	.097	.066
Hedonism (*α* = .78)	−.022	−.247	−.038	−.065	−.079	−.154
Achievement (*α* = .74)	−.136	−.157	.096	−.037	.009	−.035
Power (*α* = .68)	−.239	−.223	−.224	−.357***	−.085	−.328***
Security (*α* = .69)	−.184	−.139	−.150	−.336***	−.212	−.264**
Tradition (*α* = .64)	.017	.150	−.066	−.100	.019	.053
Conformity (*α* = .71)	−.051	−.095	−.057	−.253**	−.086	−.105
Universalism (*α* = .81)	−.064	.255	−.074	.239**	.195	.257**
Benevolence (*α* = .73)	.138	.221	.059	.258**	.090	.223**
Self‐direction (*α* = .67)	.336**	.139	.263	.329***	.122	.234**

Note

*α* = Cronbach's alpha.

*** *p* < .001;

** *p* < .01. Significance levels are Bonferroni‐Holm corrected.

#### 4.2.2 Performance measures of wisdom

Participants were presented with the life‐review task from the Berlin wisdom paradigm (BWP; Baltes & Staudinger, 2000) and interviewed about two difficult events from their life (Glück & Bluck, 2013; Glück et al., 2018). For the life‐review task, participants first completed some practice tasks to get acquainted with the think‐aloud method (Staudinger, Smith, & Baltes, 1994). Then, they were presented with the so‐called life review problem: “In reflecting over their life, people sometimes realize that they have not achieved what they had once wanted to achieve. What could a person consider and do in such a situation?” They were given ten minutes to think about the problem (Glück & Baltes, 2006) and then produced their responses, which were recorded and transcribed. The transcripts were rated by 10 trained student raters, two for each component of the Berlin wisdom paradigm (factual knowledge, procedural knowledge, life‐span contextualism, value relativism, and recognition and management of uncertainty) following the BWP manual (Staudinger et al., 1994). The total score was computed as the mean across the 10 ratings (Cronbach's alpha = .85; see Glück et al., 2013, for details).

The two other performance measures were autobiographical interviews about two difficult life events. In the MORE Life Experience model of the development of wisdom, Glück and Bluck (2013) argued that wisdom manifests itself in the way participants reflect upon past challenges in their own life. To test this prediction, participants were interviewed about a difficult life event and a difficult conflict. In both cases, they were first asked to make a list of the most difficult life events/conflicts they had encountered. (Seven participants did not complete the conflict interview because they did not remember a serious interpersonal conflict.) Then, they were interviewed about the most difficult event/conflict in the list that they (a) were willing to talk about, (b) had encountered as adults, and (c) considered as no longer ongoing. They first narrated the event freely and then answered some more specific questions (how the situation had ended, how they and, in the conflict interview, their opponent had felt at the time, how they were feeling about the experience now, whether they had learned something from the experience, and if so, what). Responses were transcribed and rated by trained student raters with respect to the components of four different wisdom models: the Berlin wisdom paradigm (Cronbach's alpha_difficult event_ = .65; Cronbach's alpha_conflict_ = .76), the three‐dimensional theory of wisdom (Ardelt, 2003; Cronbach's alpha_difficult event_ = .70; Cronbach's alpha_conflict_ = .82), the Bremen wisdom paradigm (Mickler & Staudinger, 2008; Cronbach's alpha_difficult event_ = .78; Cronbach's alpha_conflict_ = .88), and the MORE life experience model (Glück & Bluck, 2013; Cronbach's alpha_difficult event_ = .75; Cronbach's alpha_conflict_ = .88). Each student rated two components from two different models; raters received internship credit for their participation. Scores for each wisdom model were computed as averages across the respective component ratings. For the current analyses, a Promax factor analysis was run across the eight means, resulting in two factors with eigenvalues above 1 that together explained 82.53% of the variance. The four ratings for the difficult life event interview had loadings between .85 and .93 on factor 1, the four ratings for the conflict interview loaded on factor 2 (.83–.95). Thus, the factor scores for the two interviews were used in the analyses; the correlation between them was .37.

#### 4.2.3 Self‐report measures of wisdom

Participants were presented with three self‐report measures of wisdom (for details see Glück et al., 2013). The Three‐dimensional Wisdom Scale (3D‐WS, Ardelt, 2003; Cronbach's alpha = .86) measures three components. The reflective dimension assesses people's willingness to take different perspectives on phenomena and on themselves. The cognitive dimension measures the desire to gain a deep understanding of issues concerning human existence. The compassionate dimension refers to a positive, caring attitude toward others. The Adult Self‐Transcendence Inventory (ASTI, Levenson, Jennings, Aldwin, & Shiraishi, 2005; Cronbach's alpha = .83) defines wisdom as self‐transcendence that develops through the stages of self‐knowledge, detachment from external sources of self, and integration of all self‐aspects. The Self‐Assessed Wisdom Scale (SAWS, Webster, 2007; Cronbach's alpha = .90) has five subdimensions: openness (being interested in alternative viewpoints and one's own inner experience), emotional regulation (being aware and able to regulate one's own and others' emotions), humor (being able to recognize irony and to use humor for stress reduction and bonding), critical life experience (having had important and difficult personal experiences), and reminiscence and reflectiveness (reflecting about and integrating past experiences).

### 4.3 Results

Table 3 shows the correlations between the wisdom measures and the dimensions of the Schwartz Value Survey. The correlations varied considerably by measure of wisdom, with six significant correlations for the 3D‐WS, five for the ASTI, one for the difficult‐event interview, and zero (at least after the Bonferroni‐Holm correction) for the three other measures. Self‐direction was positively correlated with three wisdom measures, universalism and benevolence were positively correlated with two. Two wisdom measures were also negatively correlated with power and security. Unexpectedly, none of the wisdom measures was correlated with tradition. As the results of Studies 1 and 2 might suggest a quadratic (inverse U‐shaped) relationship between tradition and wisdom, with highly wise individuals having intermediate rather than extreme positions on this value, we also tested for quadratic components in the relationship between tradition and the wisdom measures, but there was no significant quadratic component for any measure. Closer inspection of the items measuring tradition in the SVS revealed that they were somewhat heterogeneous in content and not really consistent with the definition of “tradition” as maintaining and preserving cultural, family, or religious traditions that was used in the PVQ‐RR in Study 1. Only one item referred to respecting traditions, the others were mostly about humility and devoutness (Schwartz, 1992). Interestingly, that one item (“respect for tradition [preservation of time‐honored customs])” was negatively related to the 3D‐WS (*r* = −.161, *p* = .043), the ASTI (*r* = −.195, *p* = .014), and the BWP (*r* = −.288, *p* = .006). Positive correlations were found for the item “humble (modest, selfless)” with the SAWS (*r* = .176, *p* = .027), the ASTI (*r* = .206, *p* = .010), and the conflict interview rating (*r* = .236, *p* = .032).

### 4.4 Discussion

Study 3 investigated actual relationships between value orientations and six different measures of wisdom in a sample that included 47 wisdom nominees. Based on Studies 1 and 2, we expected positive correlations between wisdom and benevolence, universalism, self‐direction, and tradition, and a negative correlation between wisdom and power. The results were mostly consistent with these predictions, but two findings seem remarkable: first, there was considerable variation across measures of wisdom, and second, there was no relationship between wisdom and tradition.

#### 4.4.1 Differences between measures of wisdom

Based on other research, we could have expected differences between performance and self‐report measures, as self‐report measures of wisdom generally have stronger relationships with other self‐report measures (Glück et al., 2013). This was clearly the case here: while one of the three self‐report measures had six and one had five significant correlations with value dimensions, only one of the three open‐ended performance measures had only one significant correlation. (Notably, some correlations with universalism, benevolence, self‐direction, and‐‐ negatively‐‐with power became insignificant only after the Bonferroni‐Holm corrections.)

One possible explanation for these results concerns method variance. Self‐report measures of wisdom have higher correlations with other self‐report measures than performance measures do (see, e.g., Glück et al., 2013). The validity of self‐report measures of wisdom has long been controversial (Brienza, Kung, Santos, Bobocel, & Grossmann, 2018; Glück, 2018; Glück et al., 2013; Kunzmann, 2019). Simply put, the main problem is that self‐report measures do not measure how wise people actually are but how wise they think they are. At least two aspects of wisdom, however, render it difficult to judge with respect to oneself. First, there is a broad consensus that wisdom includes critical self‐reflection and intellectual humility (Grossmann, 2017; Weststrate & Glück, 2017). Therefore, very wise people might actually describe themselves as less wise in a self‐report scale than not‐so‐wise, very self‐confident people. Second, wisdom manifests itself in highly difficult, emotionally challenging situations (Glück, Bluck, Baron, & McAdams, 2005). Self‐report measures, however, assess people's views of their typical behavior. Most people may be good at, for example, taking others' perspectives or utilizing their life knowledge in many everyday situations, but few of us are wise enough to do so in the face of aggressive accusations or deeply threatening information.

With respect to the current study, it seems plausible that the correlations between wisdom and values may be somewhat inflated by the fact that both the self‐report wisdom scales and value scales were assessing people's general beliefs about themselves and about what is important in life. We believe, however, that only part of our findings can be explained by method variance. After all, the third self‐report scale did not have any significant correlations with values, which suggests a more complex pattern that also involves the content of the wisdom measures. The wisdom conception underlying the 3D‐WS emphasizes compassion and the consideration of other people's perspectives (Ardelt, 2003). The ASTI is based on a definition of wisdom as self‐transcendence, that is, as perceived interconnectedness with others and the world at large (Aldwin, Igarashi, & Levenson, 2019; Koller, Levenson, & Glück, 2017). It makes sense that these two measures, which explicitly include other‐related aspects of wisdom, would be more closely related to the self‐transcendent value dimensions of benevolence and universalism than the other measures, which focus on intrapersonal aspects (the MORE interviews and the SAWS) or general wisdom (the BWP). This interpretation would also explain why self‐direction was the only value that had a third significant correlation with an open‐ended measure.

These results bring us back to the original question: wisdom measures reflect the specific wisdom conceptions of their creators, which may, in turn, to some extent reflect those researchers' own value orientations. One could say that researchers who believe that wisdom manifests itself interpersonally created measures that include compassion, perspective‐taking, or self‐transcendence, which, therefore, have higher correlations with values like benevolence or universalism. In that sense, the results of Studies 1 and 2 on people's conceptions of wisdom may actually constitute stronger evidence concerning the relationship between wisdom and values than the direct correlations found in Study 3. Moreover, the consistency between people's conceptions and actual correlations could be viewed as an important indicator of a measure's validity: perhaps those measures of wisdom that do not include any interpersonal aspects of wisdom are missing an important aspect of that complex construct. In Study 4, we used a measure of wisdom that attempts to capture the common variance across different wisdom scales (Glück et al., 2013) rather than a measure representing any specific conception of wisdom.

#### 4.4.2 Zero correlations with tradition

Studies 1 and 2 found that while people did not imagine wise individuals to score particularly high in the tradition dimension, they did imagine them to score higher than themselves. Study 3, however, did not find any relationship between wisdom and tradition; in fact, tradition had the second‐lowest mean of all value dimensions after power. This may be related to the way the tradition dimension was represented in the SVS: the only item referring to respect for traditions was worded in a rather conservative way, and the low reliability of the tradition scale suggested low internal consistency. Future research might investigate in more detail how wise individuals negotiate the tension between respect for time‐honored customs, respect for other people's or cultures' traditions, and openness to new experiences. In Study 4, we used a more differentiated measure of tradition.

## 5 STUDY 4: WISDOM, VALUE ORIENTATIONS, AND CONCEPTIONS OF WISE VALUE ORIENTATIONS IN THREE AGE GROUPS

Study 3 found that the relationships between wisdom and values were largely consistent with people's views as identified in Studies 1 and 2, but there were differences between measures of wisdom. In addition, the connection that Study 1 and 2 participants quite consistently made between wisdom and tradition was not found in Study 3, perhaps because the value scale used in Study 3 represented tradition in a highly conservative way. For these reasons, Study 4 used different measures of both wisdom and values. We used a measure of wisdom that represents the common variance across three different wisdom scales rather than any specific wisdom conception. We also used the PVQ and not the SVS to measure value orientations and added items that explicitly referred to respect for traditions, in contrast to conforming to tradition. In addition to measuring participants' wisdom and values, we also once again assessed the values they ascribed to a wise individual, combining the designs of Studies 1 to 3. We predicted that wisdom would be related to benevolence, universalism, self‐direction, and respect for traditions, both in the comparisons between participants' own values and those they ascribed to a wise person and in the actual correlations between wisdom and values. Finally, given that Study 1 had been somewhat underpowered with respect to right‐wing participants, we aimed to replicate the findings of Study 1 concerning political attitude by asking participants to indicate their political orientation and relating political orientations to the value dimensions. In addition, the Study 3 sample may have been somewhat restricted in variability. While we attempted to recruit a wide range of participants with respect to education and age, the fact that participants had to be willing to be interviewed about highly difficult life experiences may have led to an elevated level of reflectivity even among those participants who were not nominated for wisdom. Study 4 did not include any interviews.

### 5.1 Method

#### 5.1.1 Participants

Participants were recruited through a large first‐year course on developmental psychology at the University of Klagenfurt, Austria. This course is mostly taken by students of psychology and the educational sciences. Students could earn course credit both by participating in an online survey themselves and by motivating people aged either 30–50 years (“young middle age”) or 60 years and older to participate. In earlier studies, we found that while this approach obviously creates a bias toward higher education (and interest in psychology) in the youngest segment of the sample (the university students), the older participants recruited through the students come from a wide range of educational levels and socioeconomic backgrounds. Therefore, we divided the sample into two groups. Of the 166 university students (aged 18–29 years), 77.1% were women, and 86.1% had completed the Austrian/German equivalent of high school. Of the 190 other participants, 67.9% were women, and 44.9% had completed less than high school, 24.1% had completed high school, and 31.0 had a college or university degree. As in Study 1, participants were asked to report their political orientation on a 7‐point scale. Seven participants (2.0%) identified as extreme left‐wing, 57 (16.0%) as left‐wing, 77 (21.6%) as left‐leaning, 137 (38.5%) as neutral/center, 37 (10.4%) as right‐leaning, 3 (.8%) as right‐wing, and 2 (.6%) as extreme right‐wing.

### 5.2 Measures

#### 5.2.1 Brief wisdom screening scale

To measure wisdom, we used the Brief Wisdom Screening Scale (BWSS, Glück et al., 2013). The BWSS was developed using a purely empirical approach; it consists of those 21 items from the ASTI, 3D‐WS, and SAWS that had the highest correlations with a common factor extracted in a factor analysis of the three scales. Thus, it does not represent any specific conceptual model of wisdom, but best represents the common ground across three different models. In other words, it represents the common variance across the wisdom conceptions underlying the three original measures. Given that it includes nine items from the ASTI and four from the 3D‐WS, we still expected to find similar correlations as in Study 3, but the results should be somewhat less specific to any given wisdom conception. Cronbach's alpha was .80.

#### 5.2.2 Portrait Values Questionnaire including respect for traditions

As in Study 2, we used the original 40‐item version of the Portrait Values Questionnaire (German version by Schmidt et al., 2007). In order to get a more differentiated picture of the tradition dimension, however, we added three items representing the notion of respect for traditions. The original three items measuring tradition in the PVQ described a person who (1) thinks people should not ask for more than they have and be satisfied with what they have, (2) considers religious belief as important and tries hard to do what the respective religion requires, and (3) thinks it is best to do things in traditional ways and considers it as important to keep up traditional customs. To better represent respect for tradition, we added the following three items:

1. S/he thinks it is important to have respect for cultural traditions. S/he tries to show respect for other people's traditions and customs.
2. S/he believes that people should have respect for the religious faith of others. S/he tries not to criticize other people's religious convictions.
3. S/he is interested in the religious and cultural traditions of other countries. S/he tries to approach people from other cultures and learn more about their traditions.

As in Study 2, participants first filled out the PVQ concerning their own value orientations and then the BWSS. Then, they were asked to indicate their own political orientation on a 7‐point scale ranging from “extreme left‐wing” to “extreme right‐wing” as in Study 1. Afterward, they were asked to imagine a wise person. They were told that they could either think of a person they knew or just imagine a wise individual––in any case, it should be an extraordinarily wise person. They were asked to indicate that person's gender and then presented with the PVQ in the version for that gender. Then, as in Studies 1 and 2, they were asked to indicate for each item of the PVQ how similar the person described was to their wise individual. Cronbach's alphas for the PVQ dimensions ranged from .55 (tradition, original version) to .84 (achievement); Cronbach's alpha for the three additional “respect for tradition” items was .66.

### 5.3 Results

#### 5.3.1 Differences between participants' own values and those they ascribed to a wise person

Table 4 shows the means of the two groups in the PVQ dimensions. Across groups, participants imagined a wise person to be more universalistic, benevolent, and traditional (in both the original and the newly added scales) than themselves. The university students also imagined a wise person to care more about self‐direction than themselves, while the other participants saw no significant difference between themselves and the wise person in self‐direction. This was partly due to their high mean in self‐direction (the difference between the groups in self‐direction was almost significant, *t* (354) = −1.896, *p* = .059). In addition, both age groups imagined a wise person to care significantly *less* than themselves about hedonism, achievement, and power.

**Table 4:** Study 4: Descriptive statistics and comparisons of value orientations of self and wise person: University students and other participants

Value dimension	University students (*N* = 166)			Other participants (30–87 years; *N* = 190)		
	Self	Wise person	Difference (Cohen's *d*_z_)	Self	Wise person	Difference (Cohen's *d*_z_)
Stimulation	−0.197 (0.940)	−0.312 (0.943)	.112	−0.741 (1.004)	−0.742 (1.060)	.001
Hedonism	0.482 (0.729)	−0.043 (0.936)	.515***	−0.002 (0.903)	−0.582 (1.203)	.505***
Achievement	−0.104 (0.811)	−1.114 (1.174)	.776***	−0.691 (1.162)	−1.162 (1.195)	.382***
Power	−1.027 (1.007)	−1.533 (1.137)	.386***	−1.141 (1.046)	−1.274 (.990)	.362***
Security	−0.102 (0.718)	−0.016 (0.715)	−.118	0.282 (0.688)	0.039 (0.785)	.349***
Tradition (original items)	−1.062 (0.719)	−0.441 (0.881)	−.728***	−0.630 (0.823)	−0.080 (0.881)	−.355***
Tradition (new items)	0.120 (0.879)	0.572 (1.079)	−.416***	0.142 (0.908)	0.672 (0.996)	−.582***
Conformity	−0.583 (0.742)	−0.483 (0.801)	−.114	−0.360 (0.755)	−0.352 (0.802)	−.009
Universalism	0.623 (0.604)	0.935 (0.778)	−.416***	0.705 (0.632)	0.978 (0.785)	−.362***
Benevolence	0.643 (0.563)	1.041 (0.641)	−.531***	0.689 (0.594)	.995 (0.744)	−.435***
Self‐direction	0.653 (0.590)	0.817 (0.612)	−.233**	0.775 (0.612)	0.866 (0.717)	−.122

*** *p* < .001. Significance levels are Bonferroni‐Holm corrected.

#### 5.3.2 Correlations between wisdom and values

**Figure 3:**
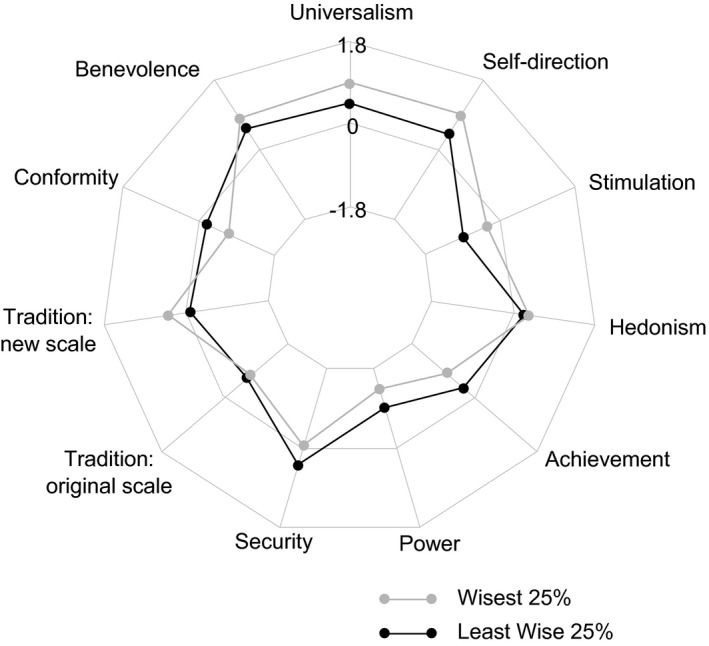
Study 4: Value orientations reported by participants in the top and bottom quartiles of wisdom

Table 5 shows the correlations between the BWSS and the dimensions of the PVQ in the two groups and in the total sample. As the table shows, the correlations were largely consistent across groups. Wisdom was most highly positively correlated with universalism (*r* = .329) and self‐direction (*r* = .321) and moderately correlated with benevolence (*r* = .225), the new respect for tradition scale (*r* = .222), and, unexpectedly, stimulation (*r* = .229). Notably, the correlations with benevolence and respect for tradition were significant only in the non‐university students. Wisdom was *negatively* correlated with conformity (*r* = −.329) and security (*r* = −.274), and, again only in the nonstudent group, with achievement (*r* = −.217), and power (*r* = −.137). Figure 3 illustrates the relationship between wisdom and values by displaying the average value orientations of participants in the top and bottom quartile of the BWSS score distribution.

**Table 5:** Study 4: Correlations between wisdom and values in university students and other participants

	University students	Other participants	Total
	*N* = 166	*N* = 190	
Stimulation	.261**	.278***	.229***
	(*p* = .001)	(*p* < .001)	(*p* < .001)
Hedonism	.017	.122	.047
	(*p* = .826)	(*p* = .093)	(*p* = .382)
Achievement	−.170	−.206**	−.217***
	(*p* = .029)	(*p* = .004)	(*p* < .001)
Power	−.051	−.191**	−.137*
	(*p* = .515)	(*p* = .008)	(*p* = .010)
Security	−.263**	−.362***	−.274***
	(*p* = .001)	(*p* < .001)	(*p* < .001)
Tradition_original	−.021	−.137	−.056
	(*p* = .784)	(*p* = .060)	(*p* = .289)
Tradition_new	.137	.285***	.222***
	(*p* = .079)	(*p* < .001)	(*p* < .001)
Conformity	−.371***	−.338***	−.329***
	(*p* < .001)	(*p* < .001)	(*p* < .001)
Universalism	.273***	.362***	.329***
	(*p* < .001)	(*p* < .001)	(*p* < .001)
Benevolence	.119	.298***	.225***
	(*p* = .128)	(*p* < .001)	(*p* < .001)
Self‐direction	.340***	.294***	.321***
	(*p* < .001)	(*p* < .001)	(*p* < .001)

*** *p* < .001;

** *p* < .01;

* *p* < .05. Significance levels are Bonferroni‐Holm corrected.

#### 5.3.3 Differences between political orientations in the values ascribed to a wise person

**Figure 4:**
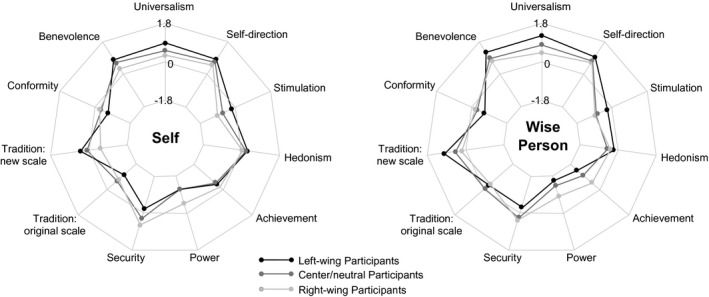
Study 4: Value orientations reported by left‐wing, center‐neutral, and right‐wing participants for themselves and a highly wise person

As Table 6 and Figure 4 show, the differences in values between left‐wing, center/neutral, and right‐wing participants were more pronounced in Study 4 than in Study 1. The three groups differed significantly in almost all value dimensions, both concerning their own values and those of a wise person. Concerning their own values, left‐wing and center‐neutral participants rated the three values of universalism, self‐direction, and benevolence highest, with some differences in order but quite similar means, and all other value dimensions considerably lower. Right‐wing participants rated security highest of all values, followed by self‐direction with a similar mean, and then, with some distance, benevolence and universalism. Concerning the values of a wise person, however, benevolence, universalism, and self‐direction were the top three in all three groups, even though the order was somewhat different. Fourth came the new respect for tradition scale in the left‐wing and center/neutral group and security in the right‐wing group. As Figure 4 shows, once again the pattern for the wise person was shifted somewhat to the top of the figure (the self‐transcendent pole), compared to the pattern for participants' own values, in all groups, but the patterns were also somewhat different between the three groups. In addition to security, right‐wing participants also imagined the wise person as higher on the values of power and achievement than left‐wing and center/neutral participants did, whereas left‐wing participants imagined the wise person as higher on the new “respect for tradition” scale and stimulation. In sum, the differences in the values that the groups with different political orientations assigned to wise individuals were along the same lines but more pronounced than in Study 1.

**Table 6:** Study 4: Means and standard deviations of left‐wing, center/neutral, and right‐wing participants in the 11 value dimensions (self and wise person) and correlations between value dimensions and political orientation

Value dimension	Left‐wing (*N* = 141)			Center/neutral (*N* = 137)			Right‐wing (*N* = 42)			Group difference+ (partial *η* ^2^)	Group difference+ (partial *η* ^2^)	*ρ*	*ρ*
	Self	Wise person	Diff. (Cohen's *d_z_*)	Self	Wise person	Diff. (Cohen's *d_z_*)	Self	Wise person	Diff. (Cohen's *d_z_*)	Self	Wise person	Self	Wise person
Self‐direction	.848 (.591)	.950 (.662)	−.144	.645 (.594)	.759 (.692)	−.176	.534 (.672)	.667 (.645)	−.166	.038**	.026*	−.188**	−.162***
										R = C. C = L	R = C. C = L		
Stimulation	−.204 (.969)	−.239 (.978)	.034	−.637 (1.009)	−.775 (1.026)	.121	−.889 (.987)	−.857 (.890)	−.030	.064***	.073***	−.247***	−.251***
										R = C < L	R = C < L		
Hedonism	.285 (.882)	−.230 (1.068)	.468***	.217 (.859)	−.478 (1.210)	.589***	.087 (.820)	−.405 (.975)	.612***	.005	.011	−.077	−.038
Achievement	−.392 (.895)	−1.417 (1.042)	.923***	−.472 (1.027)	−1.053 (1.257)	.404***	−.228 (.901)	−.512 (1.166)	.218	.007	.063***	.013	.215***
											L = C < R		
Power	−1.144 (.943)	−1.633 (.919)	.489***	−1.203 (1.005)	−1.338 (1.144)	.103	−.492 (1.156)	−.826 (1.147)	.245	.051***	.059***	.117	.211***
										L = C < R	L = C < R		
Security	−.196 (.744)	−.297 (.730)	.149	.252 (.630)	.203 (.739)	.064	.578 (.545)	.328 (.485)	.348*	.149***	.125***	.395***	.346***
										L < C < R	L < C = R		
Tradition (original)	−1.080 (.834)	−.318 (.969)	−.798***	−.627 (.707)	−.101 (.848)	−.612***	−.722 (.886)	−.387 (.851)	−.339*	.070***	.017	.215***	.046
										L < C = R			
Tradition (new)	.380 (.921)	.981 (.874)	−.667***	.105 (.743)	.466 (1.050)	−.334***	−.548 (1.018)	.167 (1.018)	−.713***	.106***	.091***	−.291***	−.286***
										R < C = L	R = C < L		
Conformity	−.665 (.753)	−.628 (.834)	−.044	−.326 (.715)	−.269 (.810)	−.069	−.234 (.787)	−.167 (.663)	−.068	.057***	.055***	.248***	.213***
										L < C = R	L < C = R		
Benevolence	.804 (.526)	1.244 (.637)	−.683***	.610 (.581)	.912 (.712)	−.362***	.331 (.674)	.714 (.765)	−.566**	.070***	.077***	−.229***	−.263***
										R < C = L	R = C < L		
Universalism	.883 (.562)	1.262 (.665)	−.637***	.548 (.579)	.831 (.793)	−.344***	.318 (.684)	.476 (.761)	−.163	.111***	.127***	−.368***	−.360***
										R < C < L	R < C < L		

*** *p* < .001;

** *p* < .01;

* *p* < .05. Significance levels are Bonferroni‐Holm corrected. *ρ* = Spearman's Rho with political orientation.

^+^ For significant group differences, Scheffé test results are reported. For example, R = C < L: the right‐wing group and center/neutral group do not differ in means, but have a significantly lower mean than the left‐wing group.

### 5.4 Discussion

The results of Study 4 largely confirmed the findings of Studies 1 to 3, but showed somewhat larger differences between the political‐orientation groups than in Study 1. Both university students and 30‐ to 87‐years‐old other participants imagined wise individuals to be high in universalism, benevolence, and self‐direction, higher than themselves both in traditionalism (the original tradition scale of the PVQ) and in respect for tradition, and low in achievement and power. When wisdom was actually measured using the BWSS, it was again significantly related to universalism, benevolence, self‐direction, and respect for tradition, but there was no relationship with traditionalism. As Figure 3 shows, participants high and low in the BWSS were equally low in traditionalism. This finding may imply that wisdom is indeed less related to a somewhat conservative, traditional orientation than people tend to think. Note, however, that in none of our studies participants imagined wise individuals to be highly traditional––they only imagined them to be somewhat more traditional than themselves.

Alternatively, these patterns suggest that the BWSS may emphasize aspects of openness to diversity and change. This notion is supported by its positive correlation with stimulation and with its negative correlations not only with achievement and (weakly) with power, but also with conformity and security. The items measuring stimulation in the PVQ refer to being interested in new experiences, taking risks, and leading an exciting life. While they are not exactly consistent with how most people would imagine a wise person, they clearly share some variance with Openness to Experience, which has frequently been identified as a predictor of wisdom both conceptually and empirically (e.g., Glück & Bluck, 2013; Staudinger, Lopez, & Baltes, 1997; Webster, 2007). How traditionally‐oriented wise individuals really are remains an open question for further research.

As mentioned earlier, the differences between the three political‐orientation groups were more pronounced than in Study 1, presumably because of the broader range of age and educational levels in Study 4. The differences in participants' own values again replicated earlier research (Caprara et al., 2006, 2008; Piurko et al., 2011; Schwartz et al., 2010). The differences in the values participants ascribed to wise persons, however, again suggest a combination of participants projecting their own values onto wise persons and some values that are generally associated with wisdom: all three political groups considered benevolence, universalism, and self‐direction as the most important values for wise individuals, although in different orders (while left‐wing participants ranked universalism highest, center/neutral and right‐wing participants ranked benevolence highest). Right‐wing participants assumed wise individuals to be higher on power and achievement than left‐wing and center/neutral participants did, which may, again, suggest that their ideas of a wise person were somewhat more along the lines of a powerful protector of his or her group than an open‐minded and humble explorer of the variability of human life (see also McAdams et al., 2008).

One clear limitation of Study 4 is that wisdom was measured by self‐report only. However, open‐ended measures such as the Berlin wisdom paradigm require an enormous effort of transcription and coding that is hardly possible with larger samples. Another important limitation is that we have no clear rationale to explain the differences between the two groups, which differ not only in age but also in education and probably in many other variables. The university students are a relatively homogeneous group, largely studying psychology and education, whereas the other participants are a broad mix of different life phases and orientations. However, as most results of this study were consistent across the two groups, their heterogeneity may actually underline the reliability of our findings.

## 6 GENERAL DISCUSSION

The research reported here started out from the question whether wisdom researchers have been projecting their own value orientations into their conceptions and measures of wisdom. While psychological wisdom theories are not very explicit about the values they associate with wisdom, at least two value‐related components of wisdom can be identified in the literature: wise individuals are assumed to care about the well‐being of others in need and to be tolerant and accepting of divergent perspectives and worldviews (see, e.g., Ardelt, 2003; Baltes & Staudinger, 2000; Glück & Bluck, 2013; Grossmann, 2017; Sternberg, 1998, 2019; Sternberg & Glück, 2019). These two value orientations are quite closely related to the moral foundations of care and fairness that, as Haidt (2008) criticized, have long been viewed as the only true cornerstones of morality by psychologists who considered their own values as the gold standard, ignoring different value systems in other cultures as well as in more conservative circles in their own culture. Therefore, the first question investigated in this paper was which values people outside academia associate with wisdom. Do people who do not share the typical worldviews of academic psychologists still share their ideas about the value orientations of wise individuals? In Studies 1, 2, and 4, participants filled out questionnaires measuring Schwartz's universal values (Schwartz, 1992, 1994, 2012; Schwartz et al., 2012) twice: once describing their own values and once describing the values of a very wise person. The second research question concerned the *actual* relationship between wisdom and value orientations: are wiser individuals indeed higher in benevolence and universalism? The answers to both questions are discussed in the following.

### 6.1 Research question 1: What value orientations do people ascribe to wise individuals?

If benevolence and universalism are universally viewed as typical of wisdom, then even people who do not endorse these values themselves should believe that wise individuals endorse them. If they are just projections of researchers' values, then those people should imagine wise individuals as being just as, or perhaps even more, nonbenevolent and nonuniversalistic than they are.

The results concerning this question were quite clear. Study 1 found that neither left‐wing nor right‐wing participants simply projected their own values onto their wisdom nominees: while all participants agreed that wise people value benevolence and self‐direction, left‐wing participants described them as more tradition‐oriented and right‐wing participants as more universalistic than themselves. Also, right‐wing participants' picture of a wise person was somewhat more authoritarian and achievement‐oriented than that of left‐wing participants. In Study 2, participants were grouped according to their actual value‐dimension scores instead of political orientations. Independent of their own values, participants described ideally wise individuals as high in benevolence, universalism, and self‐direction and low in power and achievement. They also described them as higher than themselves, though not particularly high, in tradition, and lower than themselves in hedonism. In Study 4, both university students and older, less educated participants again described wise individuals as high in benevolence, universalism, and self‐direction, low in power and achievement, and higher than themselves in tradition.

In sum, the three studies showed large commonalities in people's views across different ideologies: people believe that wise individuals are concerned about members of their own group, but also about humanity at large, that they very much appreciate the freedom to live a self‐directed life, and that they value cultural, religions, and familial traditions more than other people do. Moreover, people believe that wise individuals do not care much about self‐focused values such as power over others, personal achievement, or their personal security and pleasure. In general, even participants who held relatively strong values seemed to view wise individuals as more balanced in their perspective.

The resulting picture of a wise person is of someone who is concerned about the well‐being of others, is accepting of cultural, religious, and individual differences, respects the traditions of his or her own culture as well as those of others, and cares deeply about self‐direction, fairness and equality as fundamentals of human society. He or she does not particularly care about his or her personal security and is not at all interested in power and authority. These findings are largely in line with other research on lay theories of wisdom. Typical exemplars of wisdom, such as Mahatma Gandhi, Jesus Christ, or Martin Luther King (Paulhus, Wehr, Harms, & Strasser, 2002; Weststrate, Ferrari, & Ardelt, 2016) are individuals who engaged themselves for the well‐being of others and achieved major changes to the better by peaceful means. Our findings raise an important question: if even people far on the right side of the political spectrum consider universalism and benevolence as important characteristics of wisdom, why do they vote for populist right‐wing politicians such as (in Austria) Jörg Haider or, more recently, Sebastian Kurz or (in the U.S.) Donald Trump, who clearly do not fit into this picture of wisdom? We are grateful to an anonymous reviewer who brought up this question and proposed four possible answers: supporters of right‐wing populists may (a) consider these politicians as wise (i.e., benevolent and universalistic), or (b) see them at least as *politically* wise (which may be a subcategory with somewhat different characteristics), (c) not think wisdom is an important quality in politicians at all, or (d) think that it is not for them to decide whether something that a politician does is wise (or right) or not. This is clearly a question for future research, but our speculative response would be a combination of all four: our findings suggest that right‐wing participants consider benevolence (caring about one's own group) as more typical of wisdom than universalism (caring about a good that goes beyond one's own group). People who feel betrayed or disadvantaged may feel strongly attracted to politicians who ostensibly care about them and their group, even if it is at the expense of others. (Anecdotally, informal conversations with supporters of former governor Jörg Haider support this idea. He was considered as willing and able to protect the people of our province, which was somewhat economically disadvantaged, and he was very good at seriously taking care of the needs of people who came to him for help. Many Carinthians still tell stories of people approaching Haider at public events, receiving immediate help, and in addition, Haider remembering them and their stories years later.) Thus, people may consider populist politicians as wise or at least partially wise because they perceive them as standing up against some important injustice, or they may not see them as particularly wise but consider it as more important that someone takes care of their needs. In addition, a high degree of willingness to submit to, rather than question, authorities, even if they do something unethical, is related to right‐wing authoritarianism (Son Hing, Bobocel, Zanna, & McBride, 2007). Thus, right‐wing voters are more inclined than left‐wing voters to accept leaders' unethical behavior, arguing that they are in no position to judge their leaders or that their leaders' behavior is still a “lesser evil” compared to other dangers. Future research should look into the extent to which people of different political orientations consider wisdom as a desirable quality in politicians.

### 6.2 Research question 2: How are measures of wisdom related to value orientations?

Our findings on the values that people associate with wisdom paint a relatively clear picture. To what extent is that picture consistent with the value orientations that wise individuals actually hold? Study 3 investigated correlations between six different measures of wisdom and self‐reported value orientations in a sample that included 47 wisdom nominees. While the results showed some differences between different measures of wisdom, they were largely consistent with the findings of Studies 1 and 2: two measures of wisdom were positively related to benevolence, self‐direction, and universalism. The same was true across three different age groups in Study 4, using a different, empirically derived measure of wisdom that represents the common variance across the three self‐report measures used in Study 3. These results largely replicated earlier findings (Kunzmann & Baltes, 2003; Le, 2008; Webster, 2010). Study 4 also found a significant correlation between wisdom and the new scale measuring respect for others' religious and cultural traditions in all three age groups. Surprisingly, there was also a relationship between wisdom and stimulation, that is, a person's need for new experiences and insights, which may reflect wise individuals' desire for constant learning and growth. In sum, our findings suggest that there is a reliable relationship between wisdom and a specific set of value orientations. Wiser individuals indeed care about the well‐being of others––not just their own group, but the world at large. They value the freedom to make their own decisions and choices and have respect for cultural, religious, and individual differences.

One question that remains open concerns the relationship between wisdom and a certain amount of traditionalism or conservatism. Participants across three studies believed that wise individuals are not highly traditionalistic, but more traditionalistic than themselves. Actual correlations between wisdom and traditionalism, however, were zero across two studies. It is not yet clear whether this reflects a consistent bias across all measures of wisdom used in our research, which may overprioritize openness to change and diversity (see, e.g., Glück & Bluck, 2013; Staudinger et al., 1997; Webster, 2007), or whether it reflects a consistent bias of our participants, who may underestimate the extent to which openness is a necessary part of wisdom (see Weststrate, Bluck, & Glück, 2019, for an overview of research on people's theories of wisdom).

In sum, the actual correlations between wisdom and value orientations that we found were consistent with people's beliefs about wisdom and value orientations. Notably, however, three out of six wisdom measures in Study 3 were not significantly correlated with value orientations at all. Thus, the answer to the question that we asked in the beginning––do our conceptions of wisdom just reflect our own value orientations?––is somewhat different from what we might have expected. In fact, laypeople's conceptions of wisdom may more consistently include certain value orientations than conceptions of wisdom, or at least the measures derived from them, do. Perhaps it would be worthwhile to consider whether ethical and interpersonal aspects should be more explicitly part of our wisdom conceptions. On the other hand, it is not quite clear whether people's conceptions of wisdom should be the “gold standard” against which to assess the validity of wisdom measures. Unless they are highly wise themselves, people can only see the outside behavior of a wise person and may thus underestimate the relevance of intrapersonal aspects. In this way, wisdom conceptions are stereotypes, and stereotypes can be quite incorrect. In any case, it would seem important for future research to study the value orientations of wise individuals in more depth.

Specific limitations of the three studies were mentioned in the respective discussion sections. Here, we would like to emphasize one particularly important general limitation. All our data were collected in the German‐speaking countries, that is, in a “Western,” individualistic society. As there are clear and meaningful cultural differences in people's conceptions of wisdom (e.g., Asadi, Khorshidi, & Glück, in press; Takahashi & Overton, 2005; Yang, 2001), our findings are unlikely to be generalizable beyond “Western” countries. We hope that further research will distinguish the culturally universal and culture‐specific aspects of wisdom.

We consider the studies reported here as starting points for a broader program of research on wisdom and morality (Sternberg & Glück, 2019). Do wise individuals act in better accordance with their values than most of us do? How central is morality for wise individuals? How do wise individuals make difficult ethical decisions? If wisdom is, indeed, a universally desirable quality, we may be able to learn a lot from the way wise individuals navigate moral challenges. In today's world, it may be a good idea to listen to the wise.

## CONFLICT OF INTERESTS

The authors declared no potential conflicts of interest with respect to the research, authorship, and/or publication of this article.
